# Countermovement Jump Peak Power Changes with Age in Masters Weightlifters

**DOI:** 10.3390/sports12090259

**Published:** 2024-09-20

**Authors:** Bryan L. Riemann, Matthew Johnson, Matthew K. Helms, Andrew Hatchett, Joseph D. Vondrasek, Cullun Q. Watts, Marianne Huebner

**Affiliations:** 1 Biodynamics and Human Performance Center, Georgia Southern University—Armstrong Campus, Savannah, GA 31419, USAcw15678@georgiasouthern.edu (C.Q.W.); 2 Department of Exercise and Sport Science, University of South Carolina Aiken, Aiken, SC 29801, USA; andrewhat@usca.edu; 3 Department of Statistics and Probability, Michigan State University, East Lansing, MI 48824, USA; huebner@msu.edu

**Keywords:** sarcopenia, dynapenia, muscle power, aging, masters athletes

## Abstract

Aging is associated with decreased muscle strength and power. Power is particularly important for maintaining the independence of older adults when performing activities of daily living. The countermovement jump has been identified as a reliable and safe method to assess lower extremity power across the lifespan. The purpose of this investigation was to study sex differences and age-related changes in countermovement jump peak power among masters weightlifters with the secondary purpose of comparing results to previous reports of community and masters athletes. Female (*n* = 63, 39 to 70 yrs, med (56 yrs)) and male (*n* = 39, 35 to 86 yrs, med (59 yrs)) participants of the 2022 World Masters Championships completed three maximal effort countermovement jump repetitions following a dynamic warm-up. Vertical ground reaction forces were recorded, and peak power normalized to body mass was calculated. Results indicated significant age-related peak power among weightlifters, with the decline being significantly more pronounced in males than females. Female weightlifters exhibited less age-related decline compared to normative data as well as the other Master athlete comparison cohorts (short and long-distance runners), whereas the males demonstrated similar age-related declines as the comparison cohorts. While the female weightlifters in the current study generally demonstrated the least age-related declines in CMJ peak power of the comparative literature, the male weightlifters showed similar age-related decline rates.

## 1. Introduction

Aging is associated with sarcopenia (reduced muscle mass) [1] and sarcosthenia (reduced muscle quality and intrinsic weakening) [2], which both underpin dynapenia (decreased muscle strength and power) [3]. Collectively, these reductions hinder the ability to perform activities of daily living and reduce functional capacity, which ultimately reduces independence. Unfortunately, declines in muscle power production appear to begin at an earlier age and then increasingly decline at a more rapid rate than muscle force production [4,5,6]. Given that lower extremity muscle power is a stronger determinant of functional performance in older adults than pure muscle force production [7,8], living independently without the need for physical assistance is likely more dependent on muscle power. Hence, decrements in muscle power must be identified early so that corrective interventions can be initiated [6,9,10].

The countermovement jump (CMJ) is a reliable [11,12,13,14,15,16,17] and safe [11,18,19,20,21,22] approach to assessing lower extremity power across the lifespan. Moreover, as a weight-bearing motor task that incorporates high velocity and multi-joint contributions to move the total body center of mass, the CMJ test incorporates several key components of activities of daily living and functional performance [8,9,23,24,25,26,27]. Additionally, CMJ assessments are suggested to offer an integrated approach to evaluating the functional capability of the neuromusculoskeletal system, including voluntary motivation, bone and tendon properties (i.e., stiffness), balance, coordination, and interactions among neuromechanical components, and muscle properties (i.e., muscle mass) [18]. Multiple investigations have employed CMJ to quantify the age-related changes in lower extremity power [9,20,25,26,27,28]. By far, the most reported CMJ metric is peak power relative to body mass (W/kg). Several studies have shown that younger males exhibit higher peak power normalized to body mass than females; however, the declines in power across age are greater for males than females [9,18,25,27]. Others have shown that CMJ power is more strongly associated with age and physical functional performance than several other traditional clinical assessments such as chair rise time [26,27] and grip strength [27,29]. CMJ power better delineated individuals classified as sarcopenic compared to muscle strength [22], suggesting CMJ power may also be useful for sarcopenia screening [19,20]. Finally, diminished CMJ performance in older adults is associated with a higher risk for falling [30], vertebral fractures [31], and dysmobility syndrome [32]. Beyond peak power, a number of additional eccentric and concentric CMJ performance characteristics can be readily quantified, indicating various perspectives into lower extremity muscle function and coordination; to date, barring a few exceptions [9,24], age-related changes have largely been uninvestigated.

Large-sample randomized longitudinal interventional studies are the gold-standard study designs to understand how physical activity can influence age-related sarcopenia, sarcosthenia, and dynapenia. Unfortunately, such study designs require substantial investments of time and resources. Prior to conducting interventional studies, cross-sectional investigations of masters athletes offer a more economical alternative to exploring the potential benefits of various forms of physical activity in mitigating age-related declines in muscle strength and power. Masters athletes are typically defined as individuals over the age of 35 who actively participate in regular training routines and competitions [33]. Studying masters athletes has been described as a unique opportunity to examine biological aging effects on muscle function because they remain physically active, coupled with a lower prevalence of co-morbidities (e.g., less musculoskeletal degenerative disease and impairments). Studying masters athletes has been described as a unique opportunity to examine biological aging effects on muscle function [2,34,35]. Several investigations have examined lower extremity power via CMJ in masters runners and track and field athletes, but results are conflicting [23,35,36,37]. Those designated as power athletes (short event distance, jumpers, throwers, etc.) had higher peak power than long-distance runners [35,36,38]; but this difference in peak power varied when stratified by sex. For example, a mixed sample of male and female sprinters had significantly greater CMJ power than male and female endurance runners [36]. In contrast, male power athletes (i.e., short-distance runners and jumpers) exhibited greater CMJ power than male endurance athletes, but the difference between the two athlete groups was not statistically significant among females [37]. Age-related declines in CMJ peak power among the same masters athlete groups (power vs. endurance) are modified by sex. Michaelis et al. [35] demonstrated a lower rate of decline in peak power across age by female endurance runners compared to female short- and middle-distance runners while no significant differences were revealed between the three groups of male athletes. Conversely, two studies reported no interaction between sex and sport specialty (short-distance and jumpers, middle-distance, endurance) in masters track and field athletes [23,38]. Collectively, the above results highlight the need for further study of masters athletes to better understand sex and sport differences in age-related changes in CMJ peak power.

As a sport, weightlifting involves lifting the heaviest load possible during the snatch and clean and jerk events. Both lifts require the generation of maximal power using triple extension (i.e., hip, knee, and ankle) and motor skill coordination to raise the weighted barbell from the floor to overhead [5]. Given the movement and velocity similarity to CMJ [39] and the expectation that fast twitch muscle fibers are required for execution [34], participation in weightlifting could lessen the age-related lower extremity power declines to a greater extent than previous reports demonstrating augmented CMJ performance by masters short-distance/jumping athletes. The purpose of this investigation was to study sex differences and age-related changes in CMJ peak power among masters weightlifters. We hypothesized that (1) male weightlifters will demonstrate greater CMJ performance (peak power normalized to body mass) than females, but the age-related decline is greater in males than in females; (2) greater CMJ performance and less age-related declines compared to previous reports of community normative data; (3) CMJ performance is greater in weightlifters compared to endurance masters athletes, but similar in masters athletes in power sports.

## 2. Materials and Methods

### 2.1. Study Design

The present data were collected as part of a larger cross-sectional study examining various neuromusculoskeletal, anthropometric, arterial stiffness, and balance characteristics in masters Olympic weightlifters. Study procedures were conducted on-site at the World Masters Weightlifting Championship held in Orlando, FL, USA, 1–10 December 2022. Participants were recruited via email through their National Masters Chairs, the Masters Weightlifting Facebook site, and word-of-mouth at the competition venue. With the focus on weightlifters and the above-stated characteristics, data collection took place December 1–5, when athletes ages 45 and up were scheduled to compete.

Volunteers for study participation included 39 male (35 to 86 yrs) and 63 female (39 to 70 yrs) competitors (Table 1). All participants were void of any neuromusculoskeletal or health condition for which CMJ would be contraindicated. Participants were asked about recent food consumption, timing and servings of caffeine (1 serving = 100 mg) consumed that day, recent physical activity, and whether they took prescription medications for blood pressure, metabolic disorders, neurological disorders, and psychological disorders. All participants were informed of the testing procedures before data collection and provided written informed consent in accordance with the Declaration of Helsinki, and all study documents and procedures were approved by the institutional research ethics committee (Michigan State University STUDY00007906).

### 2.2. Countermovement Jump Testing

Previous research using CMJ testing across the lifespan has ranged from not reporting the use of any familiarization trials, having participants perform several submaximal trials, to the utilization of a separate familiarization session days prior to data collection. Given the range of methodologies, we adopted a familiarization approach that struck a balance between the extremes (i.e., we wanted participants to have several familiarization trials, but given the venue logistics, a separate familiarization session was not possible). Specifically, participants were asked to perform a brief dynamic warm-up consisting of five forward lunges on each leg and five body weight squats. Participants then completed a CMJ four-repetition gradient submaximal to maximal warm-up consisting of one CMJ at 25%, 50%, 75%, and 100% of their maximal perceived exertion. This warm-up was selected to familiarize each participant with the skill. Each CMJ was performed with feet shoulder width apart, hands akimbo, using a self-selected depth [9,24]. Following the four-repetition gradient warm-up, participants performed three maximal CMJ trials with 30 s of rest between jumps. Participants were verbally encouraged to perform each jump with maximal effort with the cue to “jump as high as you can” [9,20,26,28,35,38,40]. The same investigator (MJ) conducted all CMJ tests [23,38].

### 2.3. Data Collection and Reduction

Vertical ground reaction forces (vGRF) (1000 Hz) were captured with four force plates (PS-2142, Pasco Scientific) using the PASCO Capstone data collection software (Pasco Scientific, Roseville, CA, USA). The forceplates were located within a 1.22 m square wood frame to allow participants to perform maximally and minimize the risk of falls upon landing [41]. The forceplates were frequently calibrated (about every 2 to 4 participants) throughout data collection sessions to ensure data quality. The vGRF data were exported from the PASCO Capstone software (Version 2.7) as text files and processed using custom written MATLAB (Version 2023b) scripts (The Mathworks, Inc., Natick, MA, USA). The use of MATLAB allows for complete transparency in the data reduction process, particularly the specific criteria used to determine countermovement initiation. First, the vGRF data were summed across the four forceplates. To avoid the potential for distortion in the total body center of mass velocity and “false starts” [42,43], visual inspection of the vGRF data from each trial confirmed quiet stance prior to beginning the CMJ, followed by the manual identification of points just before and after the beginning of countermovement [43]. The computation of the vertical total body center of mass velocity was conducted beginning at the first point manually identified in quiet stance prior to the beginning of countermovement. The exact beginning of countermovement was identified by working backwards from the second point manually identified to determine the instant when the vertical total body center of mass velocity < −0.01 m∙s^−1^ [42,43]. Peak concentric power, the maximal power magnitude occurring between the start of the CMJ and ground off, was identified for each CMJ trial. Ground off was defined as the point in which the vGRF < 0.1 N∙kg^−1^. The maximal peak power value, normalized to body mass (W/kg), across the three trials was used for statistical analysis [26,36,37,44].

### 2.4. Statistical Analysis

Continuous variables were summarized with means and standard deviations and medians and ranges as appropriate. Generalized additive models for location, scale, and shape (GAMLSS) [45] were used to estimate the distribution of the CMJ peak power for a given age. GAMLSS are regression analyses that allow modeling the outcome, here CMJ performance as reflected by peak power (normalized to body mass) with a parametric distribution whose moments are estimated as smooth curves for the covariate age. We used the Box-–Cox–Cole–Green distribution of peak power. The moments for these distributions correspond to the median, coefficient of variation, and Box-–Cox power transformation needed to adjust for skewness [46]. The GAMLSS for median (*μ*), coefficient of variation (*σ*), and skewness (*ν*) with a Box-–Cox–Cole–Green distribution for the peak power is

(1) $$\begin{array}{c} \mu =\;a_{\mu }+age\times \mathrm{sex}\\ log\left(\sigma \right)=a_{\sigma }+age\upsilon =a_{\upsilon }+age \end{array}$$

We also tested non-linearity in age with penalized B-splines as the smoothing functions.

All analyses were performed using the statistical software package R version 4.3.2 [47]. GAMLSS s fitted using the R package gamlss version 5.4-12. *p*-values less than 0.05 were considered statistically significant.

Based on the similarity of measuring CMJ concentric peak power, two published studies [26,27] were chosen to address the second hypothesis based upon the requirement that eligible reports provide separate sex models with complete parameter information (slope, intercept) for peak power across age. Likewise, to address the third hypotheses, one published study [35] using similar CMJ concentric peak power measurement methods that met the same model specification criteria was identified. Because several of the previous studies being used for comparison did not restrict arm swing during CMJ performance, coupled with the documentation that arm swing augments CMJ performance [48,49,50,51,52,53], secondary comparisons were made by adjusting the current peak power data by 11%. This value was chosen because it is the most conservative augmentation estimate in the investigations considering the effects of arm swing on CMJ performance [48].

## 3. Results

### 3.1. Comparison of Female and Male Weightlifters

**Figure 1:**
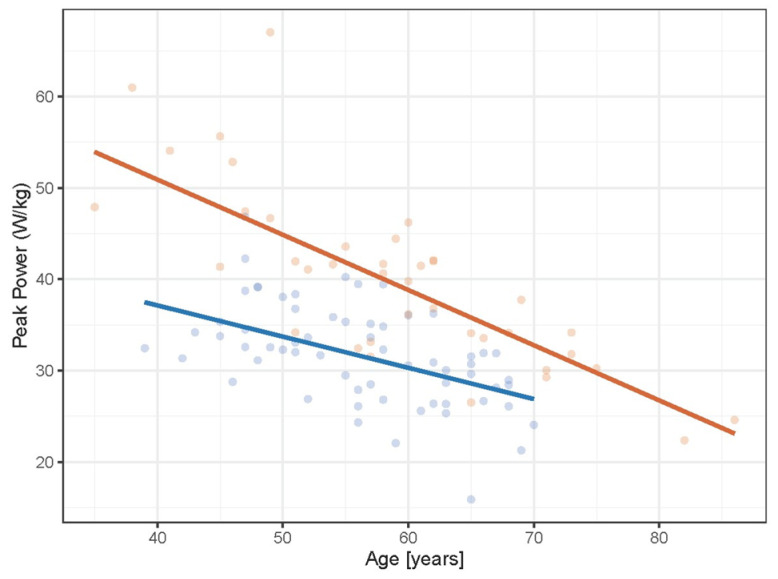
Scatterplot showing age-related declines in peak power for the female (blue) and male (orange) weightlifters.

Peak power across age decreased (Figure 1) significantly (Table 2) for both the female and male weightlifters. While the males exhibited significantly greater peak power than the females, the reduction in peak power for the males across age was also significantly greater than the females. Specifically, the percentage loss in peak power per year was 1.7% for males and 0.9% for females on average from ages 40 to 75.

### 3.2. Comparison of Weightlifters to Community Normative Data

**Figure 2:**
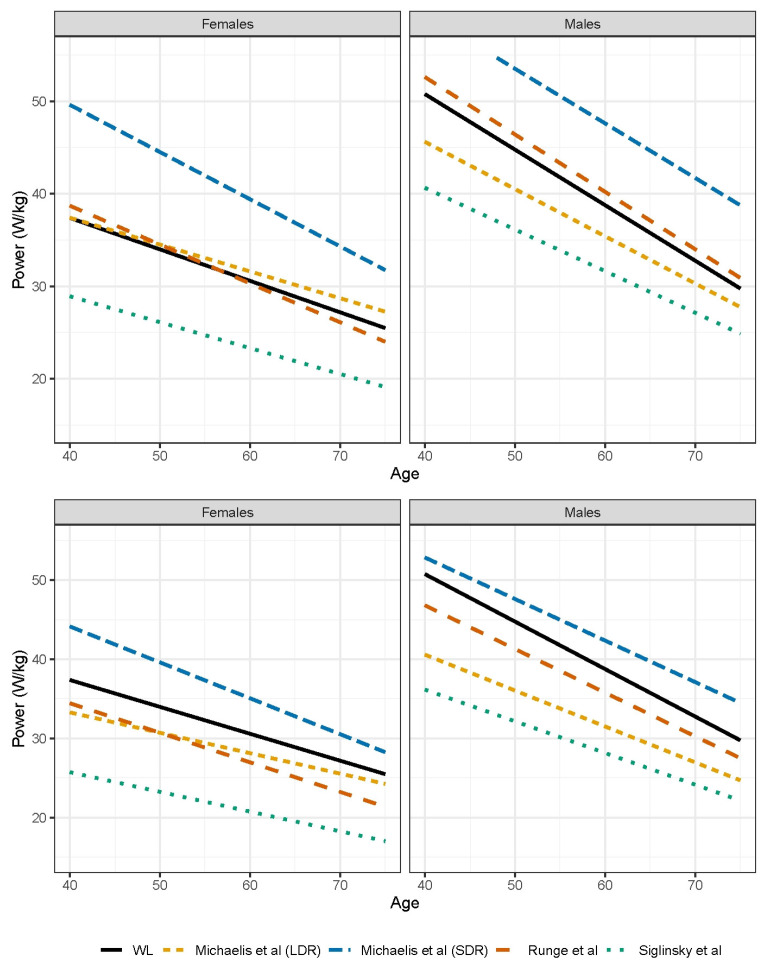
Comparison of peak power models across age between weightlifters and previous community normative (Runge et al. [26], Siglinsky et al. [27]) and masters athlete data (Michaelis et al. [35]) for the males (**left**) and females (**right**). The top plots are for the unadjusted model comparisons, while the bottom plots include the previous data being adjusted for arm swing augmentation (11%). WL: weightlifters, LDR: long-distance runners, SDR: short-distance runners.

**Figure 3:**
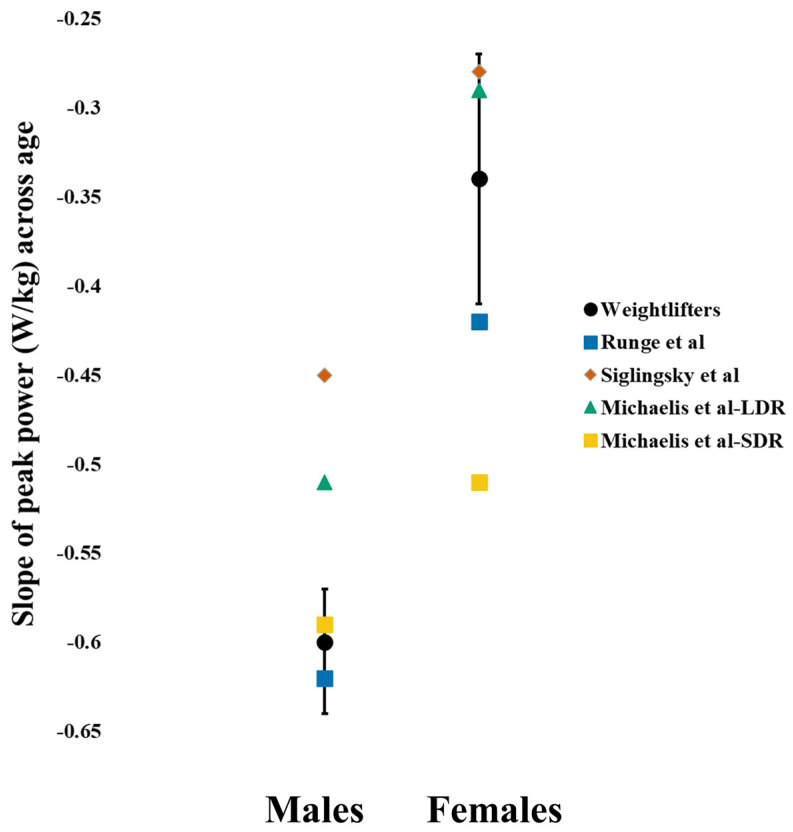
Comparison of the model slope estimates between the weightlifters and previous community normative (Runge et al. [26], Siglinsky et al. [27]) and masters athlete (Michaelis et al. [35]) data. Error bars represent 95% confidence intervals for the weightlifter estimates. More negative values indicate a steeper decline in peak power with age. LDR: long-distance runners, SDR: short-distance runners.

The comparison of the unadjusted peak powers between the weightlifters and community normative data yielded similar values to Runge et al. [26] but higher values than Siglinsky et al. [27] (Figure 2). This was consistent for both the males and females. While there appeared to be slight differences in peak power declines across age between the weightlifters and community normative data, the model slopes from the community normative data were within the 95% confidence intervals for the weightlifters (Figure 3). With the peak powers of the community normative data adjusted for arm swing, the separation between models becomes more apparent (Figure 2).

### 3.3. Comparison of Weightlifters to Masters Athletes

The comparison of the unadjusted peak powers (Figure 2) between the weightlifters and masters athletes [35] differed depending upon sex and comparison sport (long-distance runners versus short-distance runners). For the females, the short-distance runners exhibited the highest peak powers across age, whereas the long-distance runners and weightlifters were similar. Even with the arm swing adjustment, the peak power for the female short-distance runners remained higher across age. For the males, the short-distance runners also exhibited the highest peak powers, but the difference was smaller following the arm swing adjustment, particularly among the younger athletes. With the slope outside the weightlifter confidence interval (Figure 3), the decline in peak power was significantly less for the male long-distance runners compared to the weightlifters (Figure 2). The slope for male short-distance runners was nearly identical to the weightlifters. For the females, the short-distance runners exhibited significantly greater peak power declines, with their slope being outside the weightlifter slope confidence interval. The slope for the female long-distance runners was nearly identical to the weightlifters.

## 4. Discussion

This investigation compared CMJ performance in masters Olympic weightlifters to previous reports of healthy community normative and masters athlete data. Consistent with previous literature examining community populations [26,27], as well as masters athletes [35], the male weightlifters demonstrated greater CMJ peak power than the female weightlifters. Also, like the previous reports, the sex differences in the current weightlifter cohort became smaller across age as the males showed a greater CMJ peak power decline compared to the females. While the female weightlifters in the current study generally demonstrated the least age-related declines in CMJ peak power of the comparative literature, the male weightlifters showed similar age-related decline rates.

This paper sought to focus on lower extremity power. Thus, in contrast to previous literature allowing arm swing [23,25,35,36,37], we utilized CMJ methods that required participants to maintain hands akimbo [9,24] to eliminate arm swing augmenting CMJ performance [48,49,50,51,52,53]. Previous research examining the extent to which arm swing enhances CMJ performance ranges from 11% to 38% (median = 28%) [48,49,50,51,52,53]. To understand the CMJ peak power magnitudes displayed by the weightlifters in the context of previously reported healthy normative and masters athlete data allowing arm swing, we also made comparisons after adjusting the peak powers by the most conservative arm swing augmentation (11%). It is important to recognize that the 11% adjustment requires two inherent assumptions, namely that arm swing augments CMJ performance equally between males and females and that the augmentation remains constant across the lifespan. While it was previously reported [54] that active young adult males (30%) and females (27%) both showed enhanced CMJ with unrestricted arm swing, the benefit was slightly greater for the males (3%). Differences in upper/lower extremity strength, storage and utilization of strain energy, anthropometrics, and movement coordination were speculated as potential explanations. Whether arm swing has differential CMJ performance effects between middle and older adult male and female masters athletes, including weightlifters, remains unknown and represents an area for future CMJ research.

Across age, the comparison of the unadjusted CMJ peak power revealed male weightlifters had similar [26] or slightly higher values than the community normative data [25,27], whereas the female weightlifters were similar to the community normative data [25,26,27]. For both sexes, the CMJ peak power reported by Siglinsky et al. [27] was the lowest among the previous community normative studies [25,26] and the current investigation. This is likely explained by their study using the least stringent participant inclusion criteria. When peak power values were adjusted by the arm swing performance augmentation estimate (11%), the male and female weightlifters in the current study showed higher CMJ peak power than the comparative community normative data across ages [26,27]. As we used the most conservative arm swing augmentation estimate from the literature, given the median augmentation estimate being 28%, we speculate the CMJ peak power differences between the weightlifters and community normative data are likely greater than demonstrated in the comparison figures.

Remarkably, the masters female and male short-distance runners (<800 m) [35], even after arm swing augmentation adjustment, demonstrate substantially higher CMJ peak power than the masters male and female weightlifters. Based upon optimal CMJ execution being a similar movement pattern to both weightlifting events (i.e., snatch, clean and jerk), we originally expected the weightlifters to demonstrate superior CMJ peak power compared to all masters athlete runner groups reported by Michaelis et al. [35]. While short-distance running and weightlifting both require vigorous ankle, knee, and hip extension, there are several differences between the two sports that may explain the CMJ peak power differences. First, the goal of weightlifting is vertical displacement of the total body center of mass (TBCM) and loaded barbell. In contrast, the goal of short-distance running is horizontal displacement of the TBCM. Despite the difference in TBCM displacement goal, faster running is achieved through production of greater vertical forces against the ground [55], so running likely has a beneficial transfer on producing perpendicular support surface forces for vertical movements (i.e., CMJ). Second, short-distance running involves moving only the TBCM, whereas weightlifting requires moving the TBCM plus an external barbell load. Because CMJ execution involves just moving the TBCM, perhaps specificity explains the higher CMJ peak power exhibited by the short-distance runners. Thirdly, weightlifting is largely a concentric muscle action movement, while running involves both eccentric and concentric muscle actions. Because the CMJ involves both eccentric and concentric phases, with the eccentric events influencing the concentric phase [42,43], perhaps short-distance runners have an additional adaptative advantage for performing CMJ. Future studies using squat jumps could investigate these notions because of the lack of energy transfer between eccentric and concentric phases. Future research is also needed to confirm CMJ peak power differences between various masters power athletes as well as identify the etiologies (e.g., training adaptations, self-selection bias, etc.) for the differences identified. Furthermore, future research should include direct comparisons of eccentric CMJ characteristics, as well as more detailed concentric characteristics, between weightlifters and other masters athletes. Finally, it is important to remember the short-distance runners were not restricted in using arm swing when performing the CMJ. There is a possibility that because arm swing plays a contributing role in the start phase of a sprint [56], the CMJ arm swing augmentation is more pronounced in short-distance runners than the conservative 11% estimate we used when making comparisons to the weightlifters. This concept also represents an additional future research recommendation.

Previous CMJ investigations of community residing adults have reported peak power declines of 40 to 50% from the third to ninth decade [9,25,26], and baring one exception [28], the peak power decline is greater for males compared to females. Our data also support greater power declines in males (1.7%) across age than females (0.9%). There are several suggested mechanisms explaining the greater power losses in males compared to females. First, the larger absolute and relative amounts of muscle mass in males during their youth could facilitate larger absolute and relative losses of muscle and power over time [57]. Additionally, the reductions in muscle mass with age have been attributed to greater type II muscle fiber atrophy [58]. Young adult males tend to have higher proportions of type II muscle fibers, the fiber type more responsible for generating muscle strength and power, whereas females are described as having higher proportions of type I fibers [59]. The duality of males starting with a higher number of type II fibers and type II fibers being more prone to age-related atrophy might therefore explain the higher CMJ peak power declines in males compared to females. Finally, hormonal changes may also contribute to muscle strength and power declines with age. In males, testosterone levels begin to decline gradually after the age of 30, with more pronounced decreases occurring in the later decades of life [60]. Low testosterone levels have been associated with decreased muscle mass, diminished muscle strength, and increased frailty in older men [61]. For females, the decline in estrogen levels during menopause is associated with changes in muscle mass, muscle quality, and muscle strength [62]. Declines in progesterone and testosterone levels during menopause may also further exacerbate age-related declines in muscle strength and function in females. Progesterone has been shown to have anabolic effects on muscle tissue, promoting muscle protein synthesis and hypertrophy [63]. Testosterone, though present in lower levels in women compared to men, contributes to muscle strength and power output. The interplay between anabolic and catabolic hormones ultimately determines the net balance of muscle protein turnover and influences muscle mass and strength. Age-related hormonal changes tip this balance towards catabolism, contributing to the decline in strength observed with aging. The regulation of muscle mass and strength by hormones involves complex signaling pathways and interactions between endocrine, paracrine, and autocrine factors. The balance between anabolic and catabolic signaling pathways is tightly regulated under normal physiological conditions. However, disruptions in hormone levels or signaling pathways can perturb this balance, leading to alterations in muscle mass and strength.

Our hypothesis that the weightlifters would demonstrate less age-related declines in CMJ peak power than community normative data and masters runner athletes only held for the females. Based upon comparisons of the regression coefficients, the CMJ peak power declines for the male weightlifters were similar to one community study [26] and masters short-distance runners [35]. Their decline was greater than two other community studies [9,27] and masters long-distance runners [35]. This was an unexpected finding given the benefits of weightlifting on CMJ performance in young adults [64]. Our result is seemingly consistent with a previous study comparing muscle performance of male masters weightlifters (40–87 yrs) to aged-matched healthy, untrained individuals [5]. By assessing lower extremity peak power using an inertial loading device, a novel movement task for both the weightlifter and comparison participants, Pearson et al. (2002) revealed that although the weightlifters demonstrated higher peak power, the age-related declines were very similar (~1.2% decline per year). In contrast to the males, but supporting our hypothesis, the female weightlifters exhibited the lowest rate of age-related declines among the studies used for comparison [9,26,27,35]. While the rationale for our hypotheses was that weightlifting would preserve or enhance lower extremity power production, we have to acknowledge that the age of starting weightlifting relative to median female cohort age and years of experience could suggest a self-selection bias.

Recently, Hong et al. [20] proposed CMJ peak power thresholds of 19.0 W/kg for females and 23.8 W/kg for males to detect the presence of either sarcopenia or dysmobility syndrome in older adults (≥65 yrs). In the current investigation of masters weightlifters ≥65 yrs, 7.7% (1/13) and 8.3% (1/12) of the females and males, respectively, exhibited CMJ peak power below the proposed thresholds. Among all weightlifters, 1.6% (1/63) and 2.6% (1/39) met the Hong et al. [20] proposed thresholds. It is important to recognize that the CMJ methods used by Hong et al. [20] did not restrict arm swing, so their peak powers would be expected to be higher than the akimbo methods used in the current study. While different methodologies exist to define sarcopenia, meta-analyses of international data reveal estimates of sarcopenia that range from 10% to 27% [65]. The lower prevalence in the current weightlifter cohort supports the notion that studying masters athletes offers the ability to more purely examine the biological aging effects on muscle function because of their lower prevalence of co-morbidities (e.g., musculoskeletal degenerative disease and impairments) [2,34,35].

As with any cross-sectional study, the current study has several limitations. First, it is likely that a strong participant selection bias (i.e., more powerful individuals may gravitate to weightlifting) [35] exists among the participants in the current investigation. Both the self-reported weightlifting starting age and years of weightlifting experience particularly support this notion more for the females than the males based on a later starting age (49 versus 26 yrs) and fewer years (6.5 versus 25 yrs) of experience. One confounding factor with comparing our weightlifter cohort to the community normative and masters athlete data are differences in the age composition of the samples. In our cohort, based on the eligibility criteria of being a masters weightlifter, the youngest male was 35 years old and the youngest female was 37 years old. In contrast, the two community normative studies used for comparison had minimal eligible age limits of 25 years [27] and 18 years [26]. The extent of how a wider range of ages might influence the reported models used for comparisons is unknown. Additionally, other than being sufficiently proficient in weightlifting to qualify for the world championship, we are unable to control for sex or age-related differences in exercise, physical activity, and other living habits [9]. We speculate that the activity levels of our participants are likely more similar than a cross-sectional study of a general community population. Furthermore, there are potentially other aging related changes in isolated ankle, knee, and hip function such as range of motion (i.e., mobility) and strength that could influence CMJ performance. These changes could differ between masters athlete groups (i.e., weightlifting requires large ankle, knee, and hip range of motion compared to other sports) and general community populations. The potential relevancy of ankle, knee, and hip range of motion relates to countermovement depth. Common between the current investigation and previous comparison studies [26,27,35] was the use of self-selected countermovement depth. While countermovement depth can influence CMJ performance, the potency of the effect has varied between investigations [43,66,67,68]. We speculate that because of other strategy changes that can occur within the eccentric phase of the CMJ, as well as the concentric phase, the role between CMD and CMJ performance is complicated and requires a comprehensive investigational approach. Such a focus was beyond the concentration of the current hypotheses regarding age-related changes in CMJ peak power between various masters athletes and community populations but represents an area for future research. Furthermore, assessment of body composition characteristics such as lean body mass, leg muscle mass, and fat mass might also assist with explaining sex and population differences in CMJ peak power across age. Thus, it is recommended that future research comparing CMJ performance between various populations consider including assessment of isolated ankle, knee, and hip musculoskeletal characteristics as well as body composition characteristics. Finally, although there were several other studies that had considered CMJ performance in masters athletes, we were limited in our ability to make comparisons to the current cohort because separate sex analyses were not considered, full model coefficients were not provided in the reports, and small sample sizes were utilized. We encourage future research considering CMJ performance in masters athletes to conduct separate sex analyses and report full model coefficients.

## 5. Conclusions

In conclusion, consistent with previous reports of community populations as well as masters athletes, the male weightlifters demonstrated greater CMJ peak power than the female weightlifters, and the sex differences became smaller across age because males showed a greater CMJ peak power decline compared to the females. While the female weightlifters in the current study generally demonstrated the smallest age-related declines in CMJ peak power of the comparative literature, the male weightlifters showed similar age-related decline rates. These results demonstrate that adults participating in a power sport still exhibit age-related declines of lower extremity power.

## Figures and Tables

**Table 1:** Characteristics of the female and male weightlifters. All values are medians (first quartile, third quartile) except for age groups (n) and >3 servings of caffeine. Test statistics and *p* values are from the Wilcoxon test.

	N	Females (N = 63)	Males (N = 39)	Test Statistic *p* Value
Age (yrs)	102	56.0 (50.0, 63.0)	59.0 (51.0, 65.5)	*p* = 0.257
35 to 39		1	2	
40 to 49		14	7	
50 to 59		25	11	
60 to 69		22	12	
70 to 79		1	5	
80 to 89		0	2	
Height (m)	102	1.60 (1.56, 1.67)	1.72 (1.63, 1.74)	*p* < 0.001
Mass (kg)	102	59.5 (52.6,70.0)	74.4 (66.8, 87.2)	*p* < 0.001
WL start age (yrs)	96	48.5 (42.2, 55.0)	25.5 (15.0, 47.5)	*p* < 0.001
WL experience (yrs)	96	6.5 (4.0, 9.0)	25.0 (6.0, 42.7)	*p* < 0.001
EX per week (hrs)	95	7.0 (6.0, 9.0)	8.0 (7.0, 9.0)	*p* = 0.816
>3 same day caffeine servings	102	2	2	

N: number of non-missing values; WL: weightlifting; EX: exercise.

**Table 2:** Generalized additive models for location, scale, and shape coefficients of model (1) for all weightlifters (N = 102).

	Estimate (SE)	*p* Value
Mu link function: identity		
intercept	50.98 (3.51)	<0.001
age	−0.34 (0.06)	<0.001
sex	23.78 (4.31)	<0.001
age × sex	−0.26 (0.07)	0.0003
Sigma link function: log		
intercept	−1.44 (0.78)	0.071
age	−0.01 (0.01)	0.505
Nu link function: identity		
intercept	−21.50 (8.00)	0.008
age	0.40 (0.14)	0.005

SE: standard error.

## Data Availability

Upon request, the corresponding author can provide access to the data presented in this study.
